# Effects of Glucagon‐Like Peptide‐1 Receptor Agonist on Bone Metabolism and the Expression of Insulin Receptor Substrate 1 in an Osteoporotic Rat Model

**DOI:** 10.1155/ije/4418640

**Published:** 2026-03-03

**Authors:** Yao Ye, Yu-ling He, Jie He, Dan-yong Yang, Ning Xia

**Affiliations:** ^1^ Geriatric Department of Endocrinology, The First Affiliated Hospital of Guangxi Medical University, Nanning, Guangxi, 530021, China, gxmu.edu.cn; ^2^ Department of Geriatric Medicine, The Fourth Affiliated Hospital of Guangxi Medical University, Liuzhou, Guangxi, China, gxmu.edu.cn

**Keywords:** exenatide, glucagon-like Peptide 1, insulin receptor Substrate 1, osteoporosis, rats

## Abstract

**Study aim:**

This study aimed to investigate the effects of the GLP‐1R agonist Exendin 4 on bone metabolism and insulin receptor Substrate 1 (IRS‐1) expression in an osteoporotic (OP) rat model.

**Methods:**

Female SD rats were ovariectomized to establish an OP model. The rats (*n* = 18) were randomized into three groups: Sham + NS, OVX + NS, and OVX + Exendin 4 groups (six rats in each group). The NS and Exendin 4 were injected via the subcutaneous route. After 12 weeks of intervention, the right femurs of the rats were removed for bone morphometric analysis by micro‐CT scanning. Furthermore, bone biomechanical tests were conducted on the left femurs in the form of three‐point bending tests. Besides, the bilateral tibias were removed for bone tissue sectioning, HE staining, and detection of IRS‐1 expression via immunohistochemical staining. Moreover, the serum calcium and phosphorus levels were determined using a fully‐automatic biochemical analyzer. The serum levels of bone metabolism‐related parameters were analyzed using ELISA kits.

**Results:**

(1) The OVX + NS group showed a significant decrease in the number and thickness of trabeculae, fractured bone scaffold structure, and multiple adipocytes in the bone marrow cavity. Moreover, the trabeculae of the OVX + Exendin 4 group were increased in number and thickness. In addition, the bone scaffold structure was restored, and the bone marrow cavity showed fewer adipocytes than the OVX + NS group. And, the OVX + Exendin 4 group showed significantly increased BMD, BV/TV, Tb.Th, Tb.N, and bone biomechanical parameter levels (*p* < 0.05) and significantly decreased SMI and serum levels of BALP, OCN, and CTX‐1 (*p* < 0.05). (2) Immunohistochemical staining revealed that the OVX + Exendin 4 showed significantly increased expression of IRS‐1 compared with the OVX + NS group (*p* < 0.05).

**Conclusion:**

Exendin 4 exerts its antiosteoporotic effects in OVX rats by improving BMD, restoring bone microstructures, elevating bone biomechanical parameter levels, decreasing bone turnover rate, and increasing the expression of IRS‐1 in osteoblasts.

## 1. Introduction

Osteoporosis (OP) is a common metabolic bone disease characterized by degeneration of the bone microstructure, decreased bone mass, and increased risk of fractures. Bone fractures are the most common complications of OP. OP is now a global health challenge due to an increase in the aging population. Bone remodeling is a dynamic equilibrium between the formation of new bones by osteoblasts and the resorption of old bones by osteoclasts [1]. However, bone regeneration is regulated by various factors.

In recent years, the intestinal hormone glucagon‐like Peptide 1 (GLP‐1) has been shown to play an important role in the regulation of bone growth and reconstruction [2, 3]. GLP‐1 is an incretin that not only regulates blood glucose but also retards gastric emptying, suppresses appetite, promotes weight loss, has cardiovascular and neuroprotective effects, and promotes bone formation [4]. GLP‐1 is a 30 amino acids peptide hormone secreted by the intestinal L cells [5]. Endogenous GLP‐1 is not clinically useful as it is rapidly degraded by the dipeptidyl peptidase‐4 enzyme. Therefore, various GLP‐1 receptor (GLP‐1R) agonists were developed. Exenatide is a synthetic form of Exendin 4, with 53% homology to the amino acid sequence of GLP‐1. Yamada et al. reported that the GLP‐1R knockout (GLP‐1R‐/‐) mouse model showed decreased cortical bone mineral density (BMD), increased bone fragility, more osteoclasts, and increased bone resorption than the control group [6].

In an animal study, GLP‐1 R attenuated the decline in femoral and vertebral bone mass in Wistar rats fed a high‐fat/high‐calorie diet [7]. Ma et al. demonstrated in OVX rats that GLP‐1 R agonists promote bone formation by increasing the OPG/RANKL ratio and upregulating the expression of key osteoblast markers, including alkaline phosphatase (ALP), osteocalcin (OCN), Type I collagen (Col‐1), and runt‐related transcription Factor 2 (Runx2) [8]. Furthermore, Kim et al. found in Type 2 diabetic OLETF rats that GLP‐1 R agonists exert beneficial effects by reducing sclerostin levels and elevating OCN levels [9]. Besides, recent studies showed that the GLP‐1R agonists could exert their anti‐OP effects through the GLP‐1 R/PI3 K/AKT [10], GLP‐1 R/MAPK [11], and Wnt/β‐catenin [12] pathways. In addition, these studies suggested that GLP‐1R agonists could be used to manage elderly diabetic patients with OP or at an increased risk of fractures.

Meng et al. found that Exendin 4 increased bone mass and promoted bone formation in a rat model of disuse‐induced bone loss [13]. Additionally, another study demonstrated that Exendin 4 enhanced bone strength and prevented the deterioration of trabecular microstructure in aged rats with ovariectomy (OVX)‐induced OP [8]. Previous studies showed that the binding of Exendin 4 and GLP‐1R could regulate the PK A/PI3 K/AKT/GSK3 b and PKA/β‐catenin signaling pathways, thus suppressing the adipogenic differentiation of bone mesenchymal stem cells (BMSCs), facilitate osteogenic differentiation, increase the expression of the mitogen‐activated protein kinase (MAPK) signaling molecules, and enhance the proliferation and differentiation of murine preosteoblasts MC3 T3‐E1 [13, 14]. Gao et al. found that the GLP‐1R agonist, Exendin 4, could promote osteogenic differentiation and proliferation of osteoblasts, leading to bone formation [15].

The docking protein insulin receptor Substrate 1 (IRS‐1) mediates the biological actions of various cytokines and plays a central role in growth, differentiation, and cell metabolism. In bone tissues, IRS‐1 is only expressed in osteoblasts [16]. Using a gene knockout mouse model, Ogata et al. found that IRS‐1^-^/^-^ mice exhibited reductions in BMD, as well as in trabecular and cortical bone thickness. Osteoblasts deficient in IRS‐1 expression also showed decreased proliferative capacity and ALP secretion. Furthermore, the number of TRAP‐positive multinucleated osteoclasts in bone tissue was significantly reduced in IRS‐1^-^/^-^ mice [17]. These results indicate that IRS‐1 not only increases the number and activity of osteoblasts to promote bone formation but also enhances osteoclast number and activity to stimulate bone resorption. Thus, IRS‐1 plays a key role in maintaining bone remodeling balance. A previous study showed that IRS‐1 could promote bone formation and mineralization [18].

Recent statistics reveal that one in every three women and one in every five men aged over 50 years suffer from OP. Postmenopausal OP in women is the most commonly seen OP type and is characterized by diminished bone growth, increased bone resorption, and decreased bone mass due to a decrease in postmenopausal estrogen [19]. The OVX rat model has been widely adopted to investigate postmenopausal OP [20]. In the present study, an OVX rat model was established to simulate the changes in OP‐related bone metabolism under a physiological state. Therefore, this study aimed to investigate the effects of Exendin 4 on bone metabolism and IRS‐1 expression in a rat model of OP. We hope that the findings of this study will offer clinically valuable insights into the prevention and treatment of OP.

## 2. Materials and Methods

### 2.1. Experimental Animals

Eighteen female SD rats aged 6 weeks old with an average body weight of 236.54 ± 16.88 g were purchased from the Experimental Animal Center of Guangxi Medical University. All the animals were fed with standard chow and given free access to water. In addition, the animals were housed in specific pathogen‐free (SPF) animal rooms and maintained at a humidity of 50%, temperature of 25°C, and 12‐h:12‐h light‐dark cycles. The animals were acclimatized for 2 weeks. All animal experiments were approved by the Experimental Animal Ethics and Management Committee of Guangxi Medical University (No. 2018‐KY—National Natural Science Foundation—037).

### 2.2. Establishment of the OP Model and Intervention

The OP rat model was established by OVX. A total of 18 SD rats were divided into three groups (sham operation (Sham) + normal saline (NS), OVX + NS, and OVX + Exendin 4, *n* = 6 per group) using stratified randomization based on body weight. Prior to the surgery, all the rats were fasted for 12 h. However, the rats were allowed free access to water. The rats were first anesthetized by an intraperitoneal injection of 3% pentobarbital sodium (45 mg/kg). After disinfecting the skin on the back, an incision was made longitudinally from the upper margin of the top of the iliac crest to a site 1 cm below the subcostal margin and 1.5 cm beside the midline of the back. After that, the subcutaneous tissues were bluntly dissected, and the dorsal muscles were incised. The ovaries of the Sham + NS group were preserved after removing the adipose tissues near the ovaries. However, the ovaries of the OVX + NS and OVX + Exendin 4 groups were removed. Finally, the muscle layers and the skin were closed by suturing and disinfected with iodophors. After the operation, the rats received an intramuscular injection of penicillin *G* for three consecutive days as a prophylaxis against infection. And the OVX rats were allowed a 12‐week period to establish an osteoporotic condition. Following successful modeling, the OVX + Exendin 4 received a subcutaneous injection of Exendin 4 (20 μg/kg/d) for 12 weeks. However, the Sham + NS and OVX + NS groups received a subcutaneous injection of NS (20 μg/kg/d) for 12 weeks.

### 2.3. Micro‐CT Scanning of the Femur

All rats were anesthetized with an intraperitoneal injection of 3% pentobarbital sodium (45 mg/kg). The right femur of each rat was removed and fixed in 4% paraformaldehyde for 24 h. After that, bone histomorphometric analysis were conducted using a Micro‐CT scanner (Brucker Skyscan1176; pixel resolution, 8.75 μm/pixel) to visualize alterations in BMD, and bone microstructure (percent bone volume [BV/TV], trabecular thickness [Tb. Th], trabecular number [Tb. N], and structure model index [SMI]).

### 2.4. Bone Biomechanical Test

The left femurs were harvested for biomechanical testing under three‐point bending using a universal materials tester (V500 C). After identifying the null point, the mid‐diaphysis was pressed continuously at a rate of 0.01 mm/s using the universal materials tester until a femoral fracture occurred. Meanwhile, the load‐deformation curve was plotted, and the maximum load, elastic modulus, and maximum bending strength were calculated using the corresponding formulas.

### 2.5. Detection of Bone Metabolism–Related Parameters in Serum

Following the intraperitoneal injection of the anesthetic agent, abdominal aortic blood (5 mL) was taken from each rat and centrifuged (3000r/min, 15 min) to obtain the serum. Subsequently, the serum calcium and phosphorus levels were measured using a fully‐automatic biochemical analyzer. Furthermore, the serum levels of bone alkaline phosphatase (BALP) (catalog number: LV20547), OCN (catalog number: LV20549), calcitonin, and C‐terminal cross‐linked telopeptides of Type I collagen (CTX‐1) (catalog number: LV20740) were assayed using an enzyme‐linked immunosorbent assay (ELISA) kit (Animalunion Biotechnology Co., Ltd., Shanghai, China).

### 2.6. Hematoxylin and Eosin (HE) Staining for Tibial Pathohistology

The rat tibia was removed and fixed in 4% paraformaldehyde for 24 h, followed by decalcification using ethylene diamine tetraacetic acid (EDTA), which was changed regularly during the decalcification process for about 3‐4 weeks. Simultaneously, the degree of decalcification was monitored. Then, the decalcified tibial tissues were embedded in wax blocks, sectioned, fixed, and stained with HE to visualize the morphological changes of tibial tissue under a microscope.

### 2.7. Immunohistochemical Staining of IRS‐1 in Rat Tibial Tissue

The tissue wax blocks were sectioned, placed in EDTA antigen repair buffer, and washed three times using phosphate‐buffered saline (PBS). After serum blocking, the sections were incubated overnight at 4°C with the primary antibody solution of IRS‐1 (SAB, 1:200). The following day, the sections were washed thrice with PBS and incubated with secondary antibodies for 30 min at room temperature. The color was developed using diaminobenzidine (DAB), and the cell nucleus was counterstained. After that, the sections were dehydrated, blocked, and visualized under a microscope. The optical density (OD) of IRS‐1 was analyzed using the Image‐Pro Plus 6.0.

### 2.8. Statistical Analysis

Data were analyzed using the statistical software SPSS 25.0 and GraphPad Prism 8. All data were tested for normality and homogeneity of variance, and the measurement data were expressed as $\bar{x}$± s. The one‐way analysis of variance (ANOVA) was used to compare differences among multiple groups. If the variance was homogeneous, the least significance difference (LSD) tests were performed for pairwise comparisons. However, if the variance was not homogeneous, Tamhane’s T2 tests were carried out. The difference was considered statistically significant when *p* < 0.05.

## 3. Results

### 3.1. Effects of Exenatide on the Femur Microstructure and Related Parameters of OVX Rats

**FIGURE 1:**
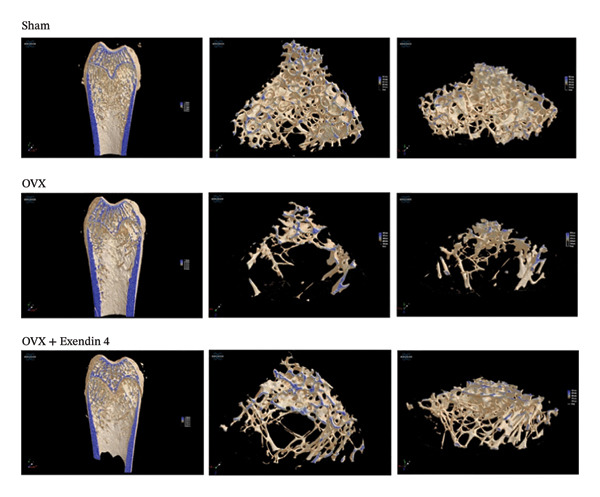
Micro‐CT images of the right femur of rats from each group.

The histomorphometric analysis for the Sham + NS group showed that the femoral metaphyseal trabeculae were tightly arranged in the same direction with a more apparent regional reticulated structure and a complete bone scaffold structure. On the other hand, the OVX + NS group showed a decrease in the number and thickness of the femoral metaphyseal trabeculae and a fractured bone scaffold structure. The OVX + Exendin 4 group showed an increase in the number of femoral metaphyseal trabeculae and restoration of the bone scaffold structure. However, the restoration of the bone scaffold structure was less than that of the Sham + NS group. Further details are illustrated in Figure 1.

**FIGURE 2:**
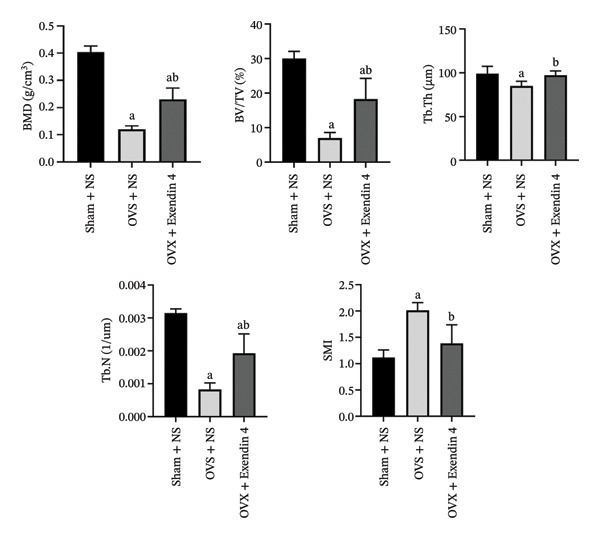
Bone microstructural parameters of the right femur of rats from each group. Note: ^a^ *p*  <  0.05 as compared with the Sham + NS group; ^b^ *p*  <  0.05 as compared with the OVX + NS group.

Bone microstructural parameters: The OVX + NS group showed significantly decreased BMD, BV/TV, Tb.Th, and Tb.N (*p* < 0.05) and increased SMI (*p* < 0.05) compared with the Sham + NS group. However, the OVX + Exendin 4 group showed an apparent increase in BMD, BV/TV, Tb.Th, and Tb.N (*p* < 0.05) and significantly decreased SMI (*p* < 0.05) compared with the OVX + NS group. Further details are demonstrated in Figure 2.

### 3.2. Effects of Exenatide on the Femur Biomechanics of OVX Rats

The OVX + NS group showed a significant decrease in the maximum load, elastic modulus, and maximum bending strength of femoral tissues (*p* < 0.05) compared with the Sham + NS group. However, the OVX + Exendin 4 group showed a significant increase in the maximum load, elastic modulus, and maximum bending strength of femoral tissues compared with the OVX + NS group (*p* < 0.05). More details are listed in Table 1.

**TABLE 1:** Effects of exenatide on femur biomechanical parameters ($\overset{¯}{x}$ ± *s*).

Group	Maximum load (N)	Elastic modulus (MPa)	Maximum bending strength (MPa)
Sham + NS	148.25 ± 16.32	80.18 ± 17.21	125.03 ± 16.45
OVX + NS	113.31 ± 20.70^a^	57.12 ± 13.18^a^	95.19 ± 11.50^a^
OVX + Exendin‐4	141.35 ± 26.85^b^	76.19 ± 16.71^b^	116.33 ± 14.88^b^

^a^ *p* < 0.05 as compared with the Sham + NS group.

^b^ *p* < 0.05 as compared with the OVX + NS group.

### 3.3. Effects of Exenatide on Serum Levels of Bone Metabolism–Related Parameters in OVX Rats

**FIGURE 3:**
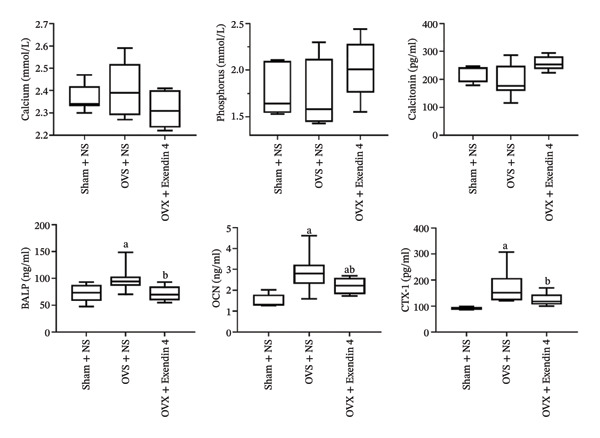
Effects of exenatide on serum levels of bone metabolism–related parameters in OVX rat. Note: ^a^ *p*  <  0.05 as compared with the Sham + NS group; ^b^ *p*  <  0.05 as compared with the OVX + NS group.

There were no significant differences in serum calcium, phosphorus, and calcitonin levels between the three groups (*p* > 0.05). However, the OVX + NS group had significantly increased serum levels of BALP, OCN, and CTX‐1 (*p* < 0.05) compared with the Sham + NS group. Furthermore, the OVX + Exendin 4 group showed significantly decreased serum levels of BALP, OCN, and CTX‐1 compared with the OVX + NS group (*p* < 0.05). More details are shown in Figure 3.

### 3.4. Tibial Pathohistological Effects of Exenatide in OVX Rats

**FIGURE 4:**
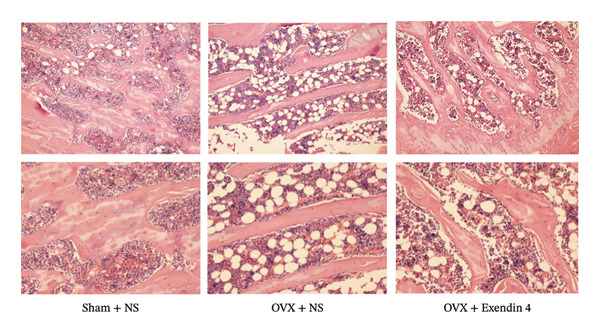
HE staining (× 100, × 200) showing the tibial tissue pathohistology of rats.

The OVX + NS group showed fewer thinner tibial trabeculae than the Sham + NS group, which were irregularly arranged with wider spacing and multiple adipocytes in the bone marrow cavity. However, the OVX + Exendin 4 group showed increased and thicker tibial trabeculae, with decreased number of adipocytes in the bone marrow cavity. More details are shown in Figure 4.

### 3.5. Effects of Exenatide on the Immunohistochemical Expression of Tibial IRS‐1 in OVX Rats

Under an electron microscope, the particles with positive IRS‐1 protein expression appeared brownish yellow or yellowish brown. A higher expression is presented in deeper colors. The OVX + NS group showed significantly decreased expression of IRS‐1 in the tibial tissues compared with the Sham + NS group (*p* < 0.05). However, the OVX + Exendin 4 group showed significantly increased expression of IRS‐1 compared with the OVX + NS group (*p* < 0.05). More details are displayed in Figure 5.

FIGURE 5Effects of exenatide on IRS‐1 expression in rat tibial tissues (× 100, × 400). Note: ^a^ *p*  <  0.05 as compared with the Sham + NS group; ^b^ *p*  <  0.05 as compared with the OVX + NS group.

**Figure:**
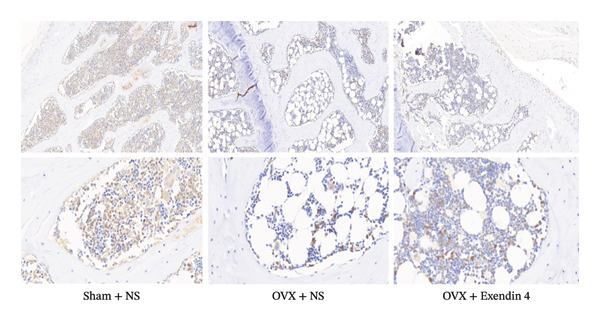
(a)

**Figure:**
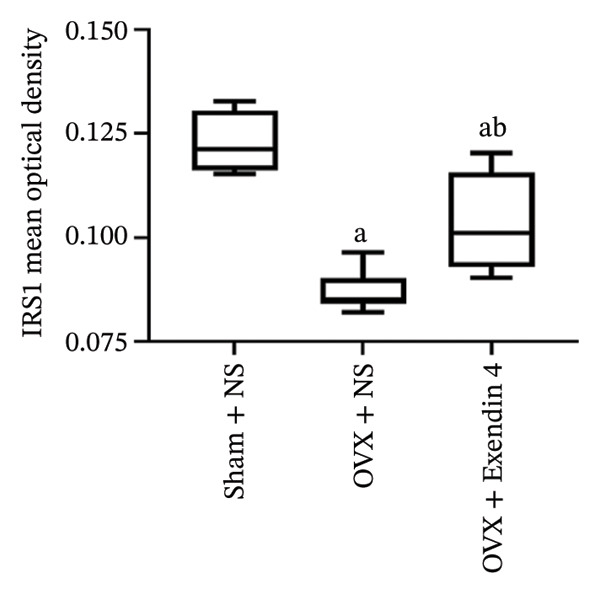
(b)

## 4. Discussion

The present study revealed that the femur of OVX rats showed a significant decrease in BMD, BV/TV, Tb.Th, Tb.N, maximum load, elastic modulus, and maximum bending strength and significantly increased SMI. The microscopic images showed successful establishment of the OP rat model, which is consistent with previous studies [21, 22]. However, some studies have reported that in OVX animal models, trabecular number decreases while trabecular thickness increases or does not decrease significantly [23, 24]. This variability may be attributed to differences in animal species, skeletal sites examined, and the time points of observation after surgery. During the rapid bone loss phase in OVX animals, bone formation activity is compensatorily enhanced to counteract increased bone resorption. While some trabeculae are lost, the remaining load‐bearing trabecular surfaces exhibit increased bone formation rates, leading to localized thickening of the preserved trabeculae. This compensatory mechanism may explain the observed phenomenon of decreased trabecular number accompanied by increased or relatively preserved trabecular thickness.

More specifically, the trabeculae of OVX rats showed a disordered arrangement with multiple adipocytes in the bone marrow cavity, indicating a decrease in the number and thickness of trabeculae and impaired bone microstructure. Moreover, the serum levels of the bone formation parameters (BALP and OCN) and bone resorption parameter (CTX‐1) were significantly increased in these rats, implying that the osteoblastic and osteoclastic activities were significantly increased due to a decrease in estrogen levels. However, OP developed as bone resorption was more pronounced than bone formation [25]. The OVX + Exendin 4 group showed increased femoral BMD, Tb.Th, Tb.N, and bone biomechanical parameters and restored the bone scaffold structure with fewer adipocytes in the bone marrow cavity, suggesting that Exendin 4 caused osteoporotic effects on OVX rats, which is consistent with previous findings [26]. Moreover, the serum levels of BALP, OCN, and CTX‐1 were significantly decreased in the OVX + Exendin 4 group, suggesting that Exendin 4 could simultaneously suppress bone formation and resorption, thus decreasing the bone turnover rate. A previous study revealed that Exendin 4 increased the osteoprotegerin/receptor activator for nuclear factor‐κB ligand (OPG/RANKL) ratio, thus inhibiting the formation of osteoclasts [8]. Furthermore, Shen et al. showed that bone resorption could be inhibited by suppressing lipopolysaccharide‐induced osteoclast formation [27]. Similarly, decreased osteoclastic activity could inhibit the enhanced bone formation, thereby lowering the high bone turnover rate resulting from the elevated osteoclast activity following OVX [21].

IRS is a key signaling protein at the postreceptor level of the insulin signaling pathway. In bone tissues, IRS‐1 is only expressed in osteoblasts and plays a vital part in bone growth, bone turnover, and fracture healing [28]. A previous study showed that [18] silencing of the IRS‐1 gene was associated with decreased expression of BALP, OCN, Col‐1, and other osteogenic parameters in MC3 T3‐E1 cells. In addition, silencing of the IRS‐1 gene was associated with decreased mineralization of MC3 T3‐E1 cells and decreased AKT phosphorylation in the IRS signaling pathway. The immunohistochemical staining in the present study showed that the Sham + NS group had increased IRS‐1 expression in the osteoblasts of femoral tissues. However, the OVX + NS group showed significantly decreased expression of IRS‐1 in osteoblasts, indicating that the low expression of IRS‐1 could be linked to OP development among OVX rats. However, administration of Exendin 4 in OVX rats resulted in increased expression of IRS‐1, suggesting that Exendin 4 could activate the IRS‐1‐mediated signaling pathways, thus increasing the expression of IRS‐1 and promoting the proliferation and differentiation of osteoblasts. However, further studies are needed to investigate the potential mechanisms behind the prevention and treatment of postmenopausal OP.

This study has several limitations. First, the sample size in the animal experiments was limited, which may introduce a certain degree of bias to the results. Second, although we demonstrated that Exendin 4 upregulates IRS‐1 protein expression in osteoblasts, the underlying molecular mechanisms within the IRS‐1 signaling pathway were not investigated.

Future studies will focus on elucidating, at the cellular and molecular levels, how Exendin 4 promotes bone formation via the IRS‐1 signaling pathway. A deeper understanding of the relationship between the IRS‐1 pathway and bone metabolism may reveal novel therapeutic targets for the clinical prevention and treatment of OP.

In conclusion, Exendin 4 exerts its anti‐OP effects on OVX rats by increasing BMD, restoring bone microstructures, elevating bone biomechanical parameter levels, decreasing bone turnover rate, and increasing the expression of IRS‐1 in osteoblasts.

## Funding

This study was funded by the National Natural Science Foundation of China (No. 8186030107).

## Ethics Statement

All animal experiments were carried out in accordance with the ethical principles of management and use of experimental animals (No. 2018‐KY—National Natural Science Foundation—037).

## Conflicts of Interest

The authors declare no conflicts of interest.

## Data Availability

Research data are not shared.
